# Deubiquitination enzyme USP35 negatively regulates MAVS signaling to inhibit anti-tumor immunity

**DOI:** 10.1038/s41419-025-07411-8

**Published:** 2025-02-27

**Authors:** Heping Zhang, Jiali Zhu, Rong He, Lin Xu, Yunfei Chen, Haihong Yu, Xuejiao Sun, Shengpeng Wan, Xiaolan Yin, Yu’e Liu, Jie Gao, Yue Li, Zhixiong Li, Yi Lu, Qing Xu

**Affiliations:** 1 Tongji University Cancer Center, Shanghai Tenth People’s Hospital, School of Medicine, Tongji University, Shanghai, China; 2 Institute for Hematologic Malignancies, East Hospital, Tongji University School of Medicine, Shanghai, China; 3 Department of Oncology, Shanghai Tenth People’s Hospital, Tongji University School of Medicine, Shanghai, China; 4 Shanghai Pharmaceuticals Holding Co Ltd, Shanghai, China; 5 Department of Hematology, East Hospital, Tongji University School of Medicine, Shanghai, China

**Keywords:** Cancer microenvironment, Cancer therapy

## Abstract

The RIG-I/MAVS signaling stimulates anti-tumor immunity by triggering the production of inflammatory cytokines. Activation of MAVS induced by viral RNA and RIG-I binding is critical in this pathway. However, the molecular mechanism underlying the regulation of MAVS activity and its function in anti-tumor immunity is not fully understood. Here, we report that the ubiquitin-specific protease 35 (USP35) negatively regulates the MAVS signaling. Mechanistically, USP35 interacts with MAVS and removes its K63-linked polyubiquitin chains, thereby inhibiting viral-induced MAVS-TBK1-IRF3 activation and downstream inflammatory gene expression. Importantly, depletion of USP35 significantly enhances the anti-tumor immunity and synergizes with oncolytic virotherapy to suppress xenograft tumor growth of melanoma cells. Thus, our study identifies USP35 as a negative regulator of MAVS signaling, representing a potential immunosuppressive factor in cutaneous melanoma.

## Introduction

Melanoma is one of the most common malignant tumors worldwide, and its morbidity and mortality are increasing annually [1]. In total, 324,635 new cases and 57,043 deaths from melanoma were registered in the GLOBOCAN 2020 database [2]. Although immunotherapy, including anti-PD-1/PD-L1 and anti-CTLA-4 antibodies, as well as oncolytic virotherapy, have emerged as the treatment for advanced or metastatic melanoma [3], a significant proportion of patients either did not respond to these therapies or developed secondary resistance [4]. Therefore, there is an urgent need to develop new therapeutic strategies for treatment efficacy.

The RIG-I-MAVS signaling pathway plays a crucial role in antiviral immunity and the anti-tumor immune response [5–7]. Sensing viral RNA or RNA analogs by RLRs (RIG-I-like receptors) results in activating and interacting with the adapter protein mitochondrial antiviral signaling protein (MAVS) to induce MAVS oligomerization [8]. Subsequently, the oligomerized MAVS activates TRAFs, which leads to the activation of interferon kinases such as TBK1 and IKKe. Then, the activated kinases phosphorylate IRF3 and IRF7, facilitating IRF3 translocation into the nucleus to stimulate the transcription of type I interferon and cytokine genes [9]. Thus, activation of MAVS is a critical step for transducing RLRs-mediated signaling. Recent studies have revealed that MAVS activity is regulated by ubiquitination [10, 11]. The E3 ubiquitin ligase TRIM31-mediated ubiquitination of MAVS is required for RIG-I-induced MAVS oligomerization and activation [12]. K27-linked ubiquitination by TRIM21 also enhances MAVS activity [13]. However, the mechanisms of deubiquitination of MAVS and its role in anti-tumor immunity are not fully understood.

Deubiquitinating enzymes (DUBs) are known to be involved in tumorigenesis and are considered potential therapeutic targets [14]. The ubiquitin-specific protease 35 (USP35) is a member of the USPs family implicated in mitophagy, cell survival, ER stress, and mitotic progression [15]. Depletion of USP35 promotes ferroptosis and suppresses lung cancer cell growth and tumor progression [16]. Of note, USP35 negatively regulates STING activity in the DNA-sensing pathway [17]. However, its role in RNA sensing and malignant melanoma remains largely unexplored.

Here, we explore the function of USP35 in MAVS signaling and find that USP35 acts as a negative regulator of MAVS-mediated type I interferon production. USP35 directly binds to MAVS and removes the K63-linked polyubiquitin chain of MAVS, a process dependent on the enzyme activity of USP35. Knockdown of USP35 combined with oncolytic virus significantly promotes the immune infiltration of CD8+ T cells and the release of IFNβ and inflammatory factors CXCL10 and CCL5, ultimately inhibiting the growth of malignant melanoma, Overall, our findings identify USP35 as a previously undescribed regulator of MAVS pathway, and targeting USP35 provides a strategy for immunotherapy of malignant melanoma.

## Materials and methods

### VSV virus

VSV-GFP was kindly provided by Professor Chengjiang Gao at Shandong University. The virus was amplified from VERO cells and stored at −80 °C. Recombinant Human Adenovirus Type 5 injection (Oncorine, H101) was sponsored by Shanghai Sanwei Biotechnology Co., Ltd.

### Plasmids

pcDNA3.1-Flag-USP35, pcDNA3.1-Flag-USP35(C450A), pcDNA3.1-Myc-USP35, pcDNA3.1-Flag-MAVS, pcDNA3.1-Flag-TBK1, pcDNA3.1-Flag-RIG-I, pcDNA3.1-HA-Ub WT, pcDNA3.1-HA-Ub-K6, K11, K27, K29, K33, K48, K63, and different truncations of USP35 and MAVS were generated by PCR and sub-cloned into the pcDNA3.1 vector. 4T-2-USP35 was constructed by PCR and sub-cloned into the 4T-2 vector. All plasmids were confirmed by DNA sequencing.

### Reagents and antibodies

The 3pRNA was purchased from Invitrogen, M2 beads (Sigma, F24265X), and ProteinA/G Plus agarose magnetic beads (Santa Cruz, P2138), and GST beads (Yisheng Biotechnology, 20507ES60) were acquired commercially. The antibodies and reagents used in this study were purchased from commercial sources: anti-Flag (Diagbio, #db7002), anti-Myc (Diagbio, #db457), anti-HA (Diagbio, #db2603), anti-USP35 (LSBio, LSC353839), anti-MAVS (Abcam, ab189109), anti-TBK1 (38066), anti-IRF 3 (CS, 4302), Rabbit anti-phospho TBK1 (Ser172) (CST, 5483), anti-phospho IRF3 (CST, 29047), anti-IgG (Abcam, ab109489), anti-GAPDH (Proteintech, 104941AP), Dual-Glo luciferase assay kit (Promega), and Glutathione Sepharose 4B (GE Healthcare).

### Construction of cell lines stably infected with shRNA and sgRNA

shRNA empty vector and a control luciferase shRNA in pLKO.1 lentiviral vector were obtained from Professor Jian Luo (East China Normal University, Shanghai, China). The shRNA targeting hUSP35 and mUSP35 were constructed by plasmid pLKO.1 and transfected by PEI or transferred by lentivirus into cells. The shRNA lentiviruses were packaged by cotransfection of the control or shRNA vector together with psPAX2 and pMD2.G into HEK293. Eight hours later, the medium was changed to fresh medium (10% FBS, 1% streptomycin–penicillin). Forty hours later, the supernatants were harvested and filtered with a 0.45 μm filter to infect B16F10, HeLa, Yummer1.7, and ID8 cells in the presence of polybrene (8 μg/mL). At twenty-four hours post-infection, cells were selected with puromycin (2–10 μg/mL) for 7 days before experiments.

We acquired the sgRNA sequence from http://cistrome.org/crispr-focus. After annealing of the foward and reverse primers, the annealed sgRNA fragment was inserted into the pLentiguide-Puro vector by molecular cloning. All plasmids were confirmed by sequencing.

### Cell culture and transfection

HEK293T, HeLa, B1610, ID8, A375, and VERO cells were purchased from the Shanghai Cell Bank of the Chinese Academy of Sciences, and YUMMER1.7 cell was purchased from ATCC. They were authenticated by STR profiling and tested for mycoplasma contamination. All cells were cultured in a 37 °C incubator containing 5% CO_2_, medium: DMEM with 10% FBS. The density of HEK293T in the plate was up to 60~70%, and transfection was performed using PEI reagent. 200 μl DMEM medium was added to a 1.5 ml sterile EP tube, and then the corresponding volume of PEI was added according to the ratio of the plasmid: PEI = 1 μg:3 μl. After 15 min, the mixture was added to a petri dish with DMEM and placed back into the incubator for further incubation.

### Real-time RT-PCR

Total RNA was extracted from cells using TRIzol reagent (Vazyme Biotech Co., Ltd) according to the manufacturer’s directions and treated with RNase-free DNase. Total RNAs (1000 ng) were used to perform the reverse transcription with HiScript II One-Step RT-PCR Kit (P611-01, Vazyme). Total RNA was extracted and assayed by real-time PCR as described with the SYBR green master mix. Primers were shown in Table S1. Data were analyzed with bio-rad software. Data were normalized by β-actin expression level in each sample. 2^−ΔΔ^Ct method was used to calculate relative expression changes.

### Co-immunoprecipitation (Co-IP) and immunoblot analysis

Transfected cells were lysed in lysis buffer (50 mM Tris-HCl (pH 7.4), 150 mM NaCl, 10% glycerol, 1 mM EDTA, 0.5% Nonidet P-40, PMSF, Na_3_VO_4_, and NaF) and cleared by centrifugation. Cleared cell lysates were incubated with 8 μl of Flag or HA beads for 3 h at 4 °C. After incubation at 4 °C, beads were washed three times with lysis buffer. The beads were then supplemented with 50 μl of protein loading buffer (2×), mixed well, and cooked at 100 °C for 5 min. Thendo the Western Blot experiments.

To detect endogenous protein interactions, cells were lysed in ice-cold lysis buffer. Cleared cell lysates were incubated with the indicated antibody and 16 µl of protein A/G beads for 3 h at 4 °C. After extensive washing, beads were boiled at 100 °C for 5 min. Proteins were resolved by SDS-PAGE and transferred onto nitrocellulose (NC) membranes (EMD Millipore), followed by immunoblotting using indicated antibodies. Immunoblots were analyzed using the Odyssey system (LI-COR Biosciences).

For western blot analysis, equal amounts of cell lysates and immunoprecipitants were resolved on a 10–12% sodium dodecyl sulfate-polyacrylamide gel electrophoresis and then transferred to an NC membrane (Cat#10639710; GE Whatman). After incubation with primary and secondary antibodies, the membranes were visualized by ECL chemiluminescence (Cat#32106; Thermo Fisher Scientific, Waltham, MA, USA).

### Luciferase reporter gene assay

The HEK293T were seeded one day earlier in 24-well plates with 2 wells for each group and transfection of each group of plasmid by PEI on the following day according to the manufacturer’s instructions. 20 ng Renila, 100 ng of IFN luciferase, 1 µg of expression plasmids including MAVS, TBK1, RIG-I, and USP35 was added to each well. Luciferase activity was measured at 24 h after transfection via the Dual-Glo Luciferase Assay System. Relative IFNβ expression was calculated as firefly luminescence relative to Renilla luminescence.

### Ubiquitination assay

In vivo deubiquitination assays were performed as described previously [18]_._ In brief, Flag-MAVS constructs were transiently transfected into 293T with or without HA-ubiquitin and USP35 WT/CA. Cells were lysed with the lysis buffer (100 μl), and the supernatants were denatured at 95 °C for 5 min in the presence of 2% SDS by lysates. The denatured lysates were diluted with lysis buffer until the concentration of SDS reduced below 0.2%, followed by immunoprecipitation (denature-IP) with the indicated antibodies. The immunoprecipitants were subject to immunoblot analysis with anti-ubiquitin, Aspirate the residual washing buffer, and boil the resin with 2× SDS loading buffer. Load samples onto SDS-PAGE gel for immunoblotting analysis. For analysis of the ubiquitination of endogenous MAVS in cells, Cells were treated with the indicated stimulation, then whole-cell extracts (denature-)were immunoprecipitated with anti-MAVS and analyzed by immunoblot with indicated antibodies.

### Protein purification and GST Pull-Down assay

The plasmids encoding GST-Vector and GST-USP35 were transformed into BL21 DE3 competent cells, which were induced with IPTG (1 mM) at 18 °C for 8 h. The cells were lysed in lysis buffer (20 mM Tris–HCl, 150 mM NaCl, 10% glycerol, and 0.5% Triton X-100).

For GST pull-down analysis, purified recombinant GST-fusion proteins were incubated with preequilibrated glutathione-Sepharose beads for 2 h followed by extensive washing. The preloaded GST resins were incubated with FLAG-MAVS lysate protein from 293 T, respectively, for 3 h at 4 °C. Precipitates were extensively washed and subjected to SDS-PAGE, followed by immunoblot analysis.

### Tumor growth and oncolytic intratumoral injection

We purchased male C57BL/6 mice aged 5–6 weeks from Shanghai Bikai Experimental Animal Company. Animals were randomly assigned to the various experimental groups. Eight mice were used per group. B16F10 were collected, and the number of inoculated cells was 5 × 10^6^ per mouse. When the tumor grew to about 6 mm, the above groups were randomly divided, and the middle of the tumor was punctured with a 1 ml sterile syringe, and H101 or PBS was injected slowly. D12, d15, d18, d21, d23, H101: 1.5 × 10^9^ vp (diluted with PBS to 50 µl) were injected intratumorally. Tumor volume was measured at 2-day intervals. Tumor volume (mm^3^) = long diameter and short diameter^2^/2, all results are expressed as mean ± SEM and analyzed by two-sample t-test. *P < 0.05, **P < 0.01, ***P < 0.001, ns, not statistically significant. When the diameter of the tumor in the control group of mice reached 15 mm, the mice were killed, and the tumors were removed.

### Flow cytometry

After tumor tissue was ground and digested by 1 mg/ml collagenase IV (Yeasen, 40510ES60) and 0.2 mg/ml DNase I (Sigma,9003-98-9) for 1 h at 37 °C, suspension cells were stained with fluorescence-labeled antibodies against Percerp-CD45, FITC-CD3, PE-CD4, APC-CD8, Percerp-CD45, FITC-CD206, PE-F4/80and CD11b. Flow cytometry was performed using BD LSRFortessa at the core facility of Tongji University, and data were analyzed with FlowJo software.

### The VSV virus and the 3pRNA activate the RNA-sensing signaling pathway

VSV was propagated and tittered by plaque assays on VERO cells. Transfection of 3p-RNA was performed according to the appropriate instructions. Samples were collected at different time points post-infection and subjected to RT-qPCR or WB assay.

### Semi-denaturing detergent agarose gel electrophoresis (SDD-AGE)

Semi-denaturing detergent agarose gel electrophoresis (SDD–AGE) was performed according to a published protocol with minor modifications [12]. Western-blot semi-dry transfer protocol was used to test the oligomerization of MAVS.

### ELISA

The concentration of IFNβ and CXCL10 was measured by Human IFNβ and CXCL10 ELISA kit (BioResearch company).

### Confocal microscopy

293T were transfected with GFP-USP35 and Flag-MAVS expressing plasmids. After 24 h, cells were stimulated for the indicated time with VSV, and then cells were fixed with 4% paraformaldehyde for 10 min at room temperature. Next, the cells were rinsed once with PBS and fixed in 4% paraformaldehyde for 15 min at room temperature. The fixed cells were permeabilized using 0.1% Triton X-100 and rinsed twice with PBS. The coverslips were blocked with blocking buffer for 1 h (0.3% BSA in PBS) and incubated in a primary antibody in blocking buffer overnight at 4 °C. Next, the coverslips were rinsed twice with blocking buffer and incubated in secondary antibodies for 1 h at room temperature in the dark. The glass coverslips were mounted using Mowiol and were examined using an LSM 510 Meta confocal system (ZEISS) under a 100× oil objective.

### Statistical analysis

All the data in this study were analyzed by GraphPad Prism 8 software and the data analysis was shown as Mean ± SEM. The T-test was used to assess pairwise statistical significance, and the one-way ANOVA test was used to assess statistical differences between multiple groups. A two-way repeated measures analysis of variance (ANOVA) was carried out to evaluate the effect of the time (before and after intervention) and the impact of time-group interaction. Where *P < 0.05, **P < 0.01, ***P < 0.001, ns is no statistical difference. Statistical analyses other than the online database were performed using R software (v4.0.2). Correlation tests were performed using personal correlation analysis.

## Results

### USP35 is highly expressed in malignant melanoma patients and correlated with tumor immune-related cell infiltration

**Fig. 1:**
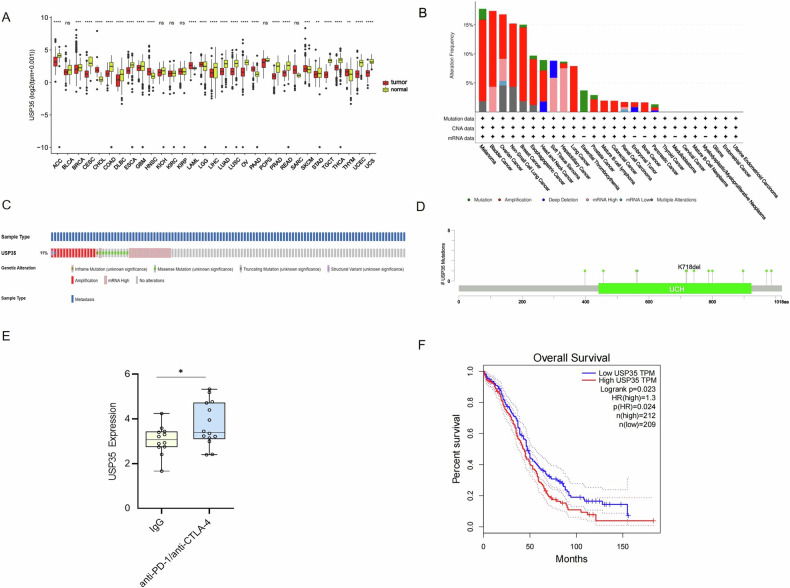
Analysis of USP35 expression in skin melanoma (SKCM) and its correlation with prognosis of tumor patients. **A** Differential expression of USP35 in tumor tissue of various origins and the corresponding adjacent tissues (data from TCGA). **B** Frequencies and copy number alterations of USP35 mutations across 26 tumor types. Alterations are color-coded as follows: green for missense mutations, red for amplifications, blue for deep deletions, orange for mRNA copy number gain, and gray for multiple alterations. Data were selected from the TCGA Pan-Cancer Atlas via cBioPortal. **C** The ratio of USP35 gene alteration found in SKCM was 11% (*n* = 363). Each example is represented as a column. The image includes all USP35 alterations found in the cohort. **D** Lollipop mutation map showing USP35 mutation sites in the SKCM cohort, which includes the location of 1 truncated mutation and 10 missense mutations in USP35. Lollipop maps are colored relative to the corresponding mutation type: missense mutations are in the green circles, and truncating mutations are in the black circles. Mutations in functional, structural domains of USP35: lollipops of the same height above the line indicate regions with segmental USP35 mutations. These data were extracted from a cohort study of 363 patients in the 2018 TCGA-SKCM Pan-Cancer Atlas (cBioPortal platform). **E** Differential expression of USP35 mRNA after IgG treatment and anti-PD1 or anti-CTLA4 treatment of malignant melanoma was analyzed using data from the GEO database. **F** Relationship between USP35 and OS in human cutaneous malignant melanoma from TCGA-SKCM data.

To explore the expression level of USP35 in pan-cancer, we first performed bioinformatics analysis using TCGA datasets and found that USP35 was significantly up-regulated in multiple tumor tissues, including melanoma and other types of cancer (Fig. 1A). Further analysis of USP35 expression in the cBioPortal database showed that USP35 had the highest proportion of genomic alterations in melanoma among 26 types of tumors, gene amplification being the most frequent alteration (Fig. 1B). Genetic alterations of USP35 were also observed in approximately 11% of cases of cutaneous melanoma in the SKCM dataset of TCGA (*n* = 40/363), including copy number amplification (*n* = 14), multiplex alterations (*n* = 14), and missense mutations (*n* = 10) (Fig. 1C). The lollipop diagram displayed that most mutations occurred in the ubiquitin carboxyl-terminal hydrolase (UCH) domain (Fig. 1D), potentially leading to truncation of USP35 and loss of its deubiquitination activity. Next, we investigated the expression level of USP35 after anti-PD-1 or CTLA-4 treatment by analyzing the data from the GEO database. The mRNA expression level of USP35 was significantly increased after anti-PD-1 or anti-CTLA-4 treatment compared with IgG treatment (Fig. 1E), suggesting USP35 may play a role in tumor immunity. We also explored the relationship between the expression level of USP35 and the overall survival (OS) of patients with melanoma using the GEPIA database. Kaplan–Meier survival curves indicated that patients with high USP35 expression levels had poorer OS (*P* = 0.038, HR = 1.3) (Fig. 1F). These findings suggest that USP35 is highly expressed in malignant melanoma tissues and is associated with poor prognosis in patients with melanoma. To assess whether USP35 is associated with the tumor microenvironment in malignant melanoma, we conducted immune infiltration estimation by six algorithms, including xCell, QUANTISEQ, MCPcounter, EPIC, CIBERSORTABS, and CIBERSORT. As shown in the bubble plot, USP35 expression was positively correlated with the immune infiltration of Treg cells, M2 macrophages, and neutrophils, and negatively correlated with infiltration of B cells, CD8^+^ T cells, CD4^+^ T cells, M1-type macrophages, and dendritic cells (Supplementary Fig. 1A). Similar results were also observed using xCELL and CIBERSORT algorithms (Supplementary 1B–G). These data suggest that the expression of USP35 was negatively correlated with the infiltration of immune cells in tumors.

### USP35 inhibits the activation of the type I interferon signaling pathway by MAVS

**Fig. 2:**
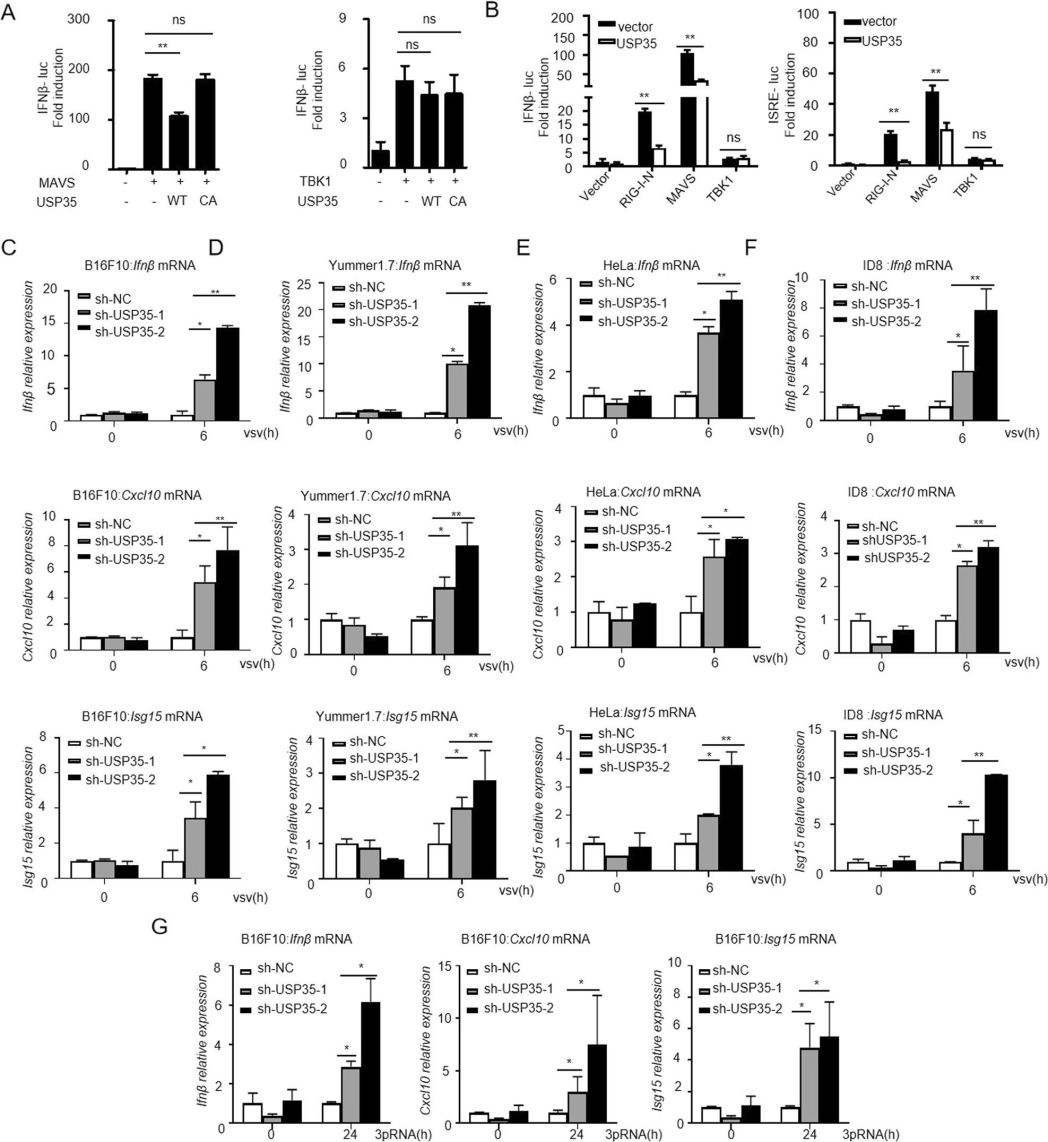
USP35 regulates MAVS activity. **A** Transfection of the indicated plasmids in 293 T to detect the effect of USP35-WT and CA on the activity of IFNβ reporter gene, *n* = 3. **B** Co-transfection of the indicated plasmids in 293T to detect overexpression of USP35 for RIG-I, MAVS, and TBK1-activated IFNβ/ISRE reporter gene, *n* = 3. Data are shown as Mean ± s.d. Significance statistics were performed by One-way ANOVA, where **P < 0.01, ns, no statistical difference, and n represents the number of experimental replicates. **C** In B16F10, a stable USP35 knockdown cell line was generated using shRNA. Cells were collected at 0 h and 6 h post-VSV virus infection, and qRT-PCR was performed to measure the mRNA levels of *IFNβ*, *CXCL10*, and *ISG15*. *N* = 3. **D**–**F** In the Yummer1.7, HeLa, and ID8 with knockdown of USP35, the levels of IFN β, CXCL10, and ISG 15 were measured by qRT-PCR after infection with VSV virus, *n* = 3. The data show that all are Mean ± s.d, the significance statistics method is One-way ANOVA, where *P < 0.05, **P < 0.01, ns is not statistically different, and n is the number of experimental replicates. **G** mRNA expression levels of IFNβ, CXCL10, and ISG15 were detected by qRT-PCR at 24 h after 3pRNA transfection in B16F10. *N* = 3. Data are shown as Mean ± s.d. Significance statistics were performed by One-way ANOVA, where *P < 0.05, ns not statistically different. *n* is the number of replicates of the experiment.

Since the RIG-I-MAVS pathway plays a key role in promoting anti-tumor T-cell infiltration, we next investigated whether USP35 regulates this signaling. Dual luciferase reporter assay displayed that ectopic expression of wild-type USP35, but not the catalytically inactive USP35-C450A mutant, inhibited MAVS-induced IFNβ luciferase reporter (IFNβ-Luc) activity (Fig. 2A). Furthermore, USP35 was found to inhibit RIG-I, but not TBK1-activated IFNβ-Luc expression (Fig. 2B), suggesting that the regulation is at the layer of RIG-I-MAVS. IFN activates the expression of interferon-stimulated genes (ISGs), which contain ISRE (interferon-stimulated response element) elements in the promoters [19]. we also found that USP35 also inhibited RIG-I or MAVS-induced ISRE-Luc activity (Fig. 2B). Next, we explored whether USP35 regulates RIG-I/MAVS-mediated expression of the interferon and related inflammatory factors. In mouse malignant melanoma cell lines B16F10 and Yummer1.7, knockdown of USP35 by shRNAs, which specifically targeted USP35 mRNA, significantly enhanced VSV virus-induced expression of inflammation and antiviral-associated factors, including IFNβ, CXCL10, and ISG15 (Supplementary Fig. 2A, B; Fig. 2C, D). Similar results were also observed in HeLa and ID8 cells (Fig. 2E, F, Supplementary Fig. 2C, D). Viral RNAs activate the innate immune receptor retinoic acid-inducible gene I (RIG-I)-like receptor (RLR). Short double-stranded RNA (dsRNA) with both 5’-triphosphate part-5’-triphosphate RNA (3p-RNA) is a specific ligand for RIG-I [20]. Upon binding to 3p-RNA, RIG-I triggers downstream signaling through IFN regulatory factor 3 (IRF3), mitogen-activated protein kinase (MAPK), and NF-κB pathways via MAVS. We used 3pRNA to activate RIGI/MAVS signaling and found that the mRNA expression level of IFNβ, CXCL10, and ISG15 was significantly enhanced by USP35 depletion (Fig. 2G). Furthermore, ELISA experiments further confirmed that the protein levels of IFN-β and CXCL10 were increased in USP35-knockdown cells (Supplementary Fig. 2H). Taken together, these findings suggest that USP35 is a negative regulator of RIG-I-MAVS signaling.

### USP35 interacts with MAVS in vivo and in vitro

**Fig. 3:**
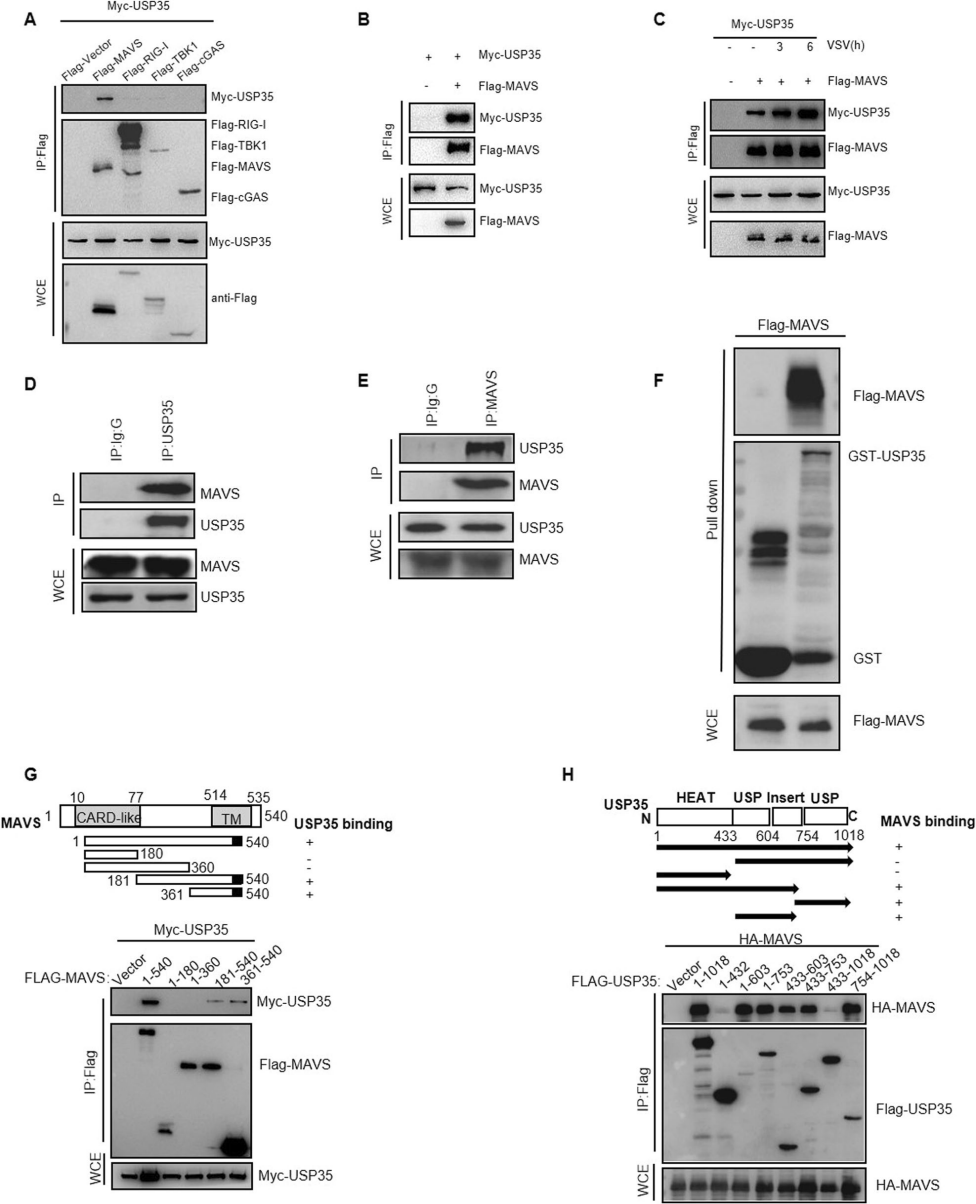
USP35 interacts with MAVS. **A** Co-transfection of Myc-USP35 with the indicated constructs in 293T. The binding of USP35 to RIGI, TBK1, cGAS, and MAVS was detected by co-immunoprecipitation (co-IP). **B** 293T were transfected with Myc-USP35 and Flag-MAVS. Co-IP was performed to detect the interaction between USP35 and MAVS. **C** Overexpression of the indicated plasmids in 293T were infected with 0.5 MOI VSV virus for the indicated time, and then the lysates were subjected to co-IP. **D** The interaction between endogenous USP35 and MAVS in ID8 was examined by co-IP experiment using anti-USP35 antibody. **E** The interaction between endogenous USP35 and MAVS in ID8 was examined by co-IP experiment using anti-MAVS antibody. **F** GST pull-down assay was performed using purified USP35 protein with Flag-tagged MAVS expressed in 293T. **G** 293T transfected with the indicated constructs were subjected to co-IP and western blot analysis. Schematic illustration of the primary structure of MAVS is shown. **H** 293T transfected with the indicated constructs were subjected to co-IP and western blot analysis. Schematic illustration of the primary structure of USP35 is shown.

To investigate the mechanism how USP35 inhibits the RIG-I-MAVS pathway, we examined the interaction between USP35 and MAVS. Co-immunoprecipitation (Co-IP) experiments in 293 T co-transfected with Myc-USP35 and Flag-tagged RIG-I, MAVS, or TBK1 showed that USP35 strongly interacted with MAVS (Fig. 3A, B). Reciprocal Co-IP assay displayed that endogenous USP35 could bind to endogenous MAVS (Fig. 3D, E). To validate whether USP35 directly interacts with MAVS, we performed a GST Pull-down assay using the purified USP35 protein and detected a direct interaction between GST-USP35 and MAVS (Fig. 3F). To explore whether the binding of USP35 to MAVS is regulated by VSV virus stimulation, we infected cells with the VSV virus. The interaction between USP35 and MAVS was enhanced in a time-dependent manner following VSV treatment (Fig. 3C). Immunofluorescence staining further confirmed the colocalization of USP35 with MAVS upon RNA infection (Supplementary Fig. 2D).

MAVS contains an N-terminal CARD-like domain and a C-terminal transmembrane domain TM [21]. To identify the domains involved in the binding of USP35 to MAVS, we constructed a series of truncations of MAVS. Co-IP experiments showed that the transmembrane domains of MAVS interacted with USP35 (Fig. 3G). Additionally, we created a series of truncated mutants of USP35 and found that MAVS could bind to the USP structural domain at the C-terminus of USP35 (Fig. 3H). Taken together, these results suggest that USP35 interacts with MAVS both in vivo and in vitro, and this interaction is regulated by viral stimulation.

### USP35 modifies MAVS through K63 chain deubiquitination

**Fig. 4:**
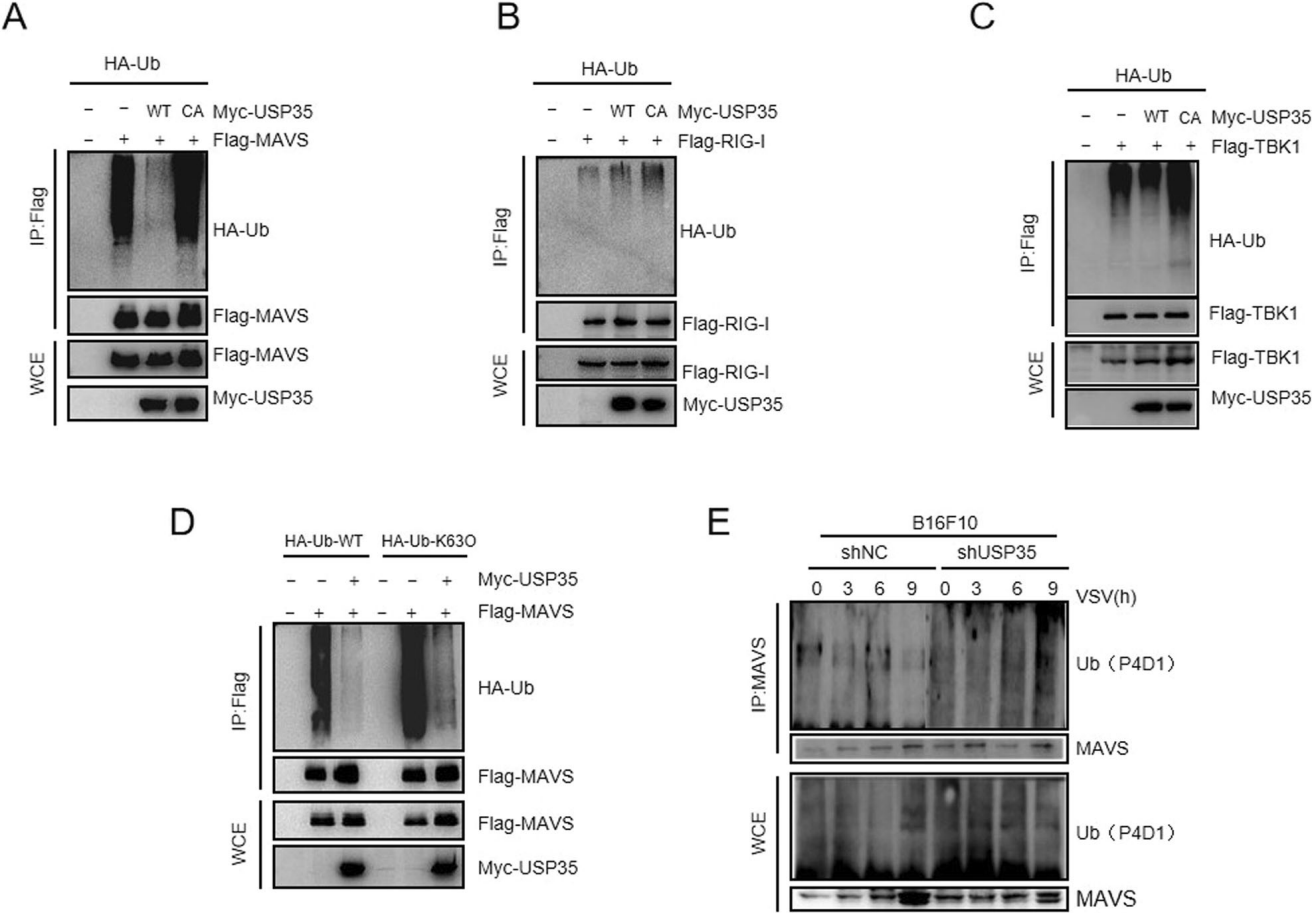
USP35 acts by removing K63 chain ubiquitination modifications on MAVS. **A** HEK293T were co-transfected with Flag-MAVS, HA-Ub, and Myc-USP35-WT or USP35-C450A plasmids. Ubiquitination of MAVS was detected by Co-IP. **B** Flag-MAVS, HA-Ub, and Myc-USP35-WT or USP35-C450A plasmids were coexpressed in HEK293T, ubiquitination of RIG-I was detected by Co-IP. **C** Co-expressed Flag-MAVS, HA-Ub, and Myc-USP35 WT or USP35 C450A plasmids in HEK293T, ubiquitination of TBK 1 was detected by Co-IP. **D** MAVS-Flag, USP35-Myc, and HA-Ub or HA-Ub K63O expression plasmids, and ubiquitination of MAVS was detected by immunoprecipitation. **E** In B16F10 shNC and shUsp35 knockdown were transfected with Flag-MAVS plasmid. Samples were collected at different time points for ubiquitination assays and western blotting to detect endogenous MAVS ubiquitination.

Since USP35 is a key member of the deubiquitination enzyme family, we explored whether USP35 regulates the ubiquitination of MAVS. The ubiquitination experiments showed that USP35-WT, but not catalytically inactive USP35-C450A mutant could deubiquitinate MAVS (Fig. 4A). In contrast, both USP35-WT and USP35-CA had no significant effect on the ubiquitination of RIG-I or TBK1 (Fig. 4B, C), indicating that USP35 specifically deubiquitinates MAVS. Moreover, the knockdown of USP35 promoted the ubiquitination of endogenous MAVS (Fig. 4E). To explore which type of polyubiquitin linkage on MAVS is targeted by USP35, we co-transfected MAVS with WT-ubiquitin or different ubiquitin variants. Our data showed that USP35 could remove K6, K11, K27, K33, K29, K48, and K63-linked polyubiquitin chains from MAVS (Supplementary Fig. 3A). Viral infection has been shown to lead to K63-type linked ubiquitination of MAVS at the mitochondrial outer membrane which subsequently induces MAVS aggregation and activation [12]. We co-expressed MAVS-Flag, USP35-Myc, and HA-Ub or HA-Ub K63O (K63 only) in 293 T. The results showed that USP35 removed the K63-type ubiquitination of MAVS (Fig. 4D). Semi-denaturing gel electrophoresis (SDD-AGE) was performed to examine MAVS multimerization. The results showed that USP35 knockdown increased the level of MAVS multimerization induced by RNA infection (Supplementary Fig. 3B). Furthermore, the half-life of MAVS protein was also not significantly changed by USP35 knockdown or overexpression. These results indicate that USP35 does not affect the stability of MAVS(Supplementary Fig. 3D, E). Together, these data suggest that USP35 is a deubiquitinase for MAVS.

### USP35 regulates the MAVS-TBK1-IRF3 signaling pathway

Next, we investigated whether USP35 regulates MAVS activity. RIG-I-MAVS signaling triggers phosphorylation and activation of TBK1/IRF3, which stimulates IRF3-mediated transcription of interferon and pro-inflammatory cytokines [22]. To explore this, we first established USP35 knockdown (shUSP35) or knockout (sgUSP35) cells (Supplementary Fig. 2A–F).

**Fig. 5:**
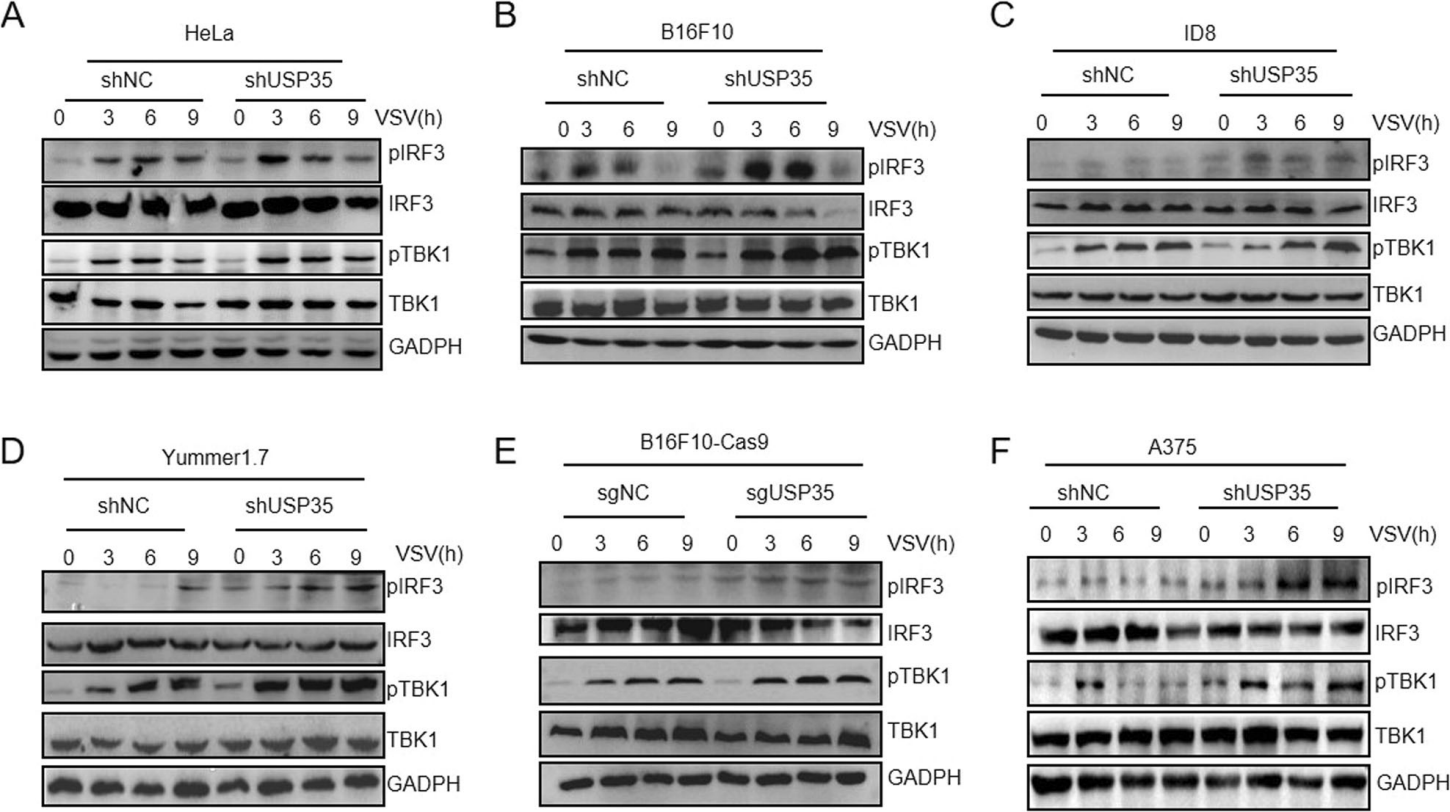
USP35 regulates the MAVS-TBK1-IRF3 signaling pathway. **A**, **B** HeLa, Yummer1.7 infected with VSV (0, 3, 6, and 9 h) in knockdown of USP35 and control groups were assayed for pTBK1, pIRF3, TBK1, IRF3 and GAPDH by western blot. **C**, **D**, **F** VSV infected with knockdown of USP35 and control groups of ID8, Yummer 1.7, A375 (0, 3, 6, and 9 h), and protein levels of pTBK1, pIRF3, as well as TBK1, IRF3, and GAPDH were detected by Western Blot. **E** USP35 was knocked out in B16F10cas9 and the knockout of USP35 and control groups were infected with VSV (0, 3, 6, and 9 h), and the protein expression of p-TBK1, p-IRF3, and GAPDH, TBK1, and IRF3 were detected by WB.

The phosphorylation levels of the TBK1 and IRF3 were detected in B16F10, A375, and Yummer1.7 at different time points after VSV stimulation. The results showed that the phosphorylation of both TBK1 and IRF3 was significantly enhanced in the shUSP35 group or the sgUSP35 group compared to control cells (Fig. 5B, D–F, Supplementary Fig. 4B, D, E, F). Similar results were observed in HeLa and ID8 with knockdown of USP35 (Fig. 5A, C, Supplementary Fig. 4A, C). Restoration of Myc-USP35 in USP35-depleted A375 inhibited TBK1 and IRF3 activation upon RNA virus stimulation (Supplementary Fig. 3C). These results suggest that USP35 inhibits MAVS activity to restrain type I interferon production. To confirm these findings, we rescued Myc-USP35-WT expression in USP35-knockdown A375 and stimulated them with RNA viruses, which were found to fail to activate TBK1 or IRF3 phosphorylation (Supplementary Fig. 3C), further supporting the role of USP35 in inhibiting MAVS activity. These results suggest that USP35 negatively regulates MAVS activity, thereby restraining type I interferon production.

### The knockdown of USP35 enhances the anti-tumor immunity of oncolytic viruses in malignant melanoma

**Fig. 6:**
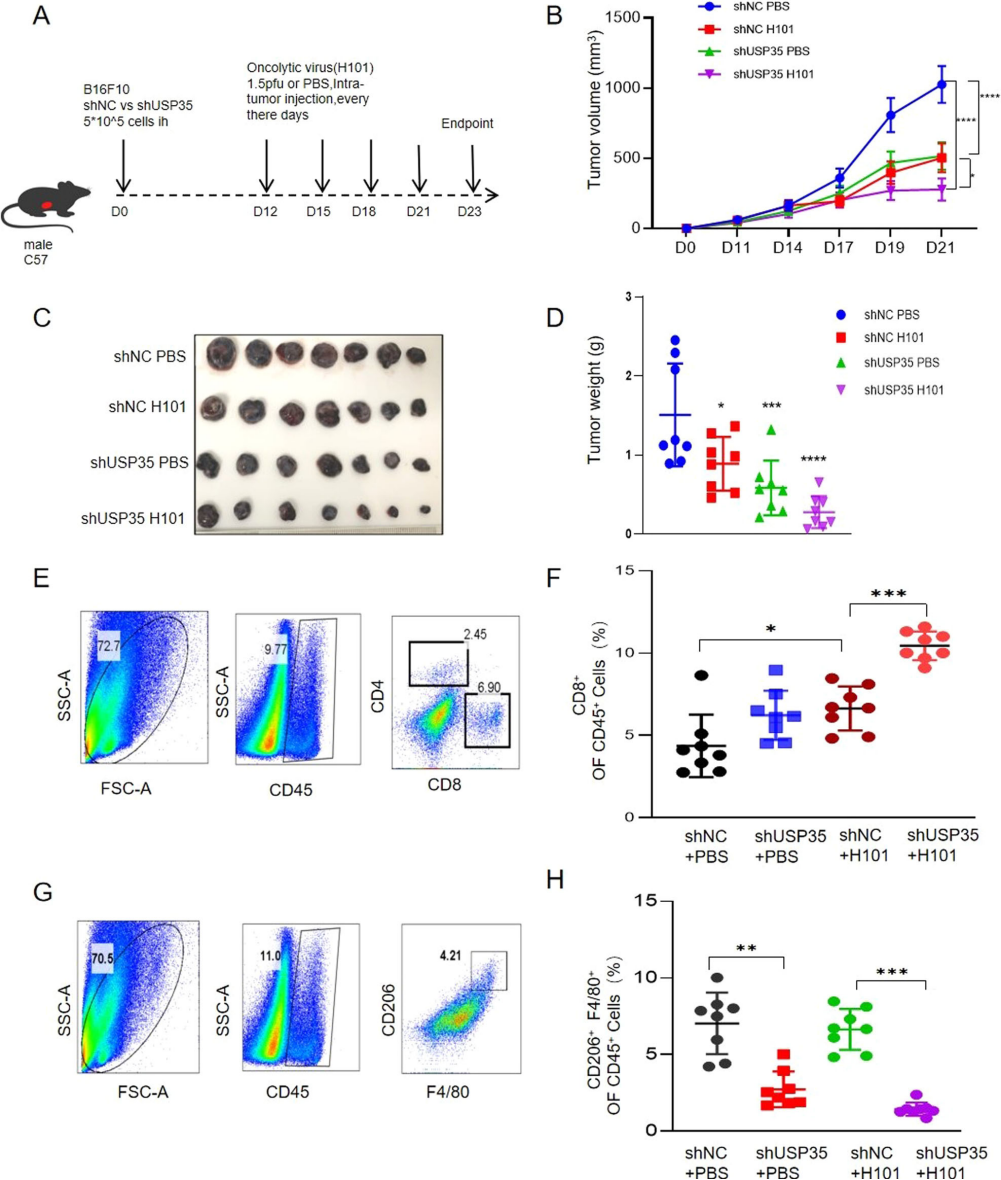
USP35 regulates immune cell infiltration in malignant melanoma and enhances the sensitivity to oncolytic virotherapy. **A** When the tumors in mice reached approximately 6 mm in size, they were injected intratumorally with tumor Oncolytic virus H101 or PBS (d12, d15, d18, d21, d23), respectively, according to the corresponding groups; H101: 1.5 × 10^9^ vp was diluted to 50 μl, while the control group received an equal volume of PBS. Eight mice were used per group. **B**, **C** When tumor length and diameter in control mice reached approximately 15 mm, the mice were executed for weighing and photographing according to groups. **D** Tumor growth was monitored by measuring the long and short diameters of tumors every 2 days. Tumor volume was calculated using the formula: volume (mm³) = (long diameter $\times$ short diameter²)/2. Tumor growth curves were plotted and subjected to statistical analysis. **E** In the B16F10 mouse model, the percentages of positive CD4+ and CD8^+^ T cells were measured in the different experimental groups. **F** The proportion of CD8^+^ T cells positive for infiltration in each group. **G** In the B16F10 mouse model, the percentages of positive F4/80^+^ and CD206^+^ macrophages were measured in the different experimental groups. **H** The proportion of CD206^+^ macrophages in each group.

**Fig. 7:**
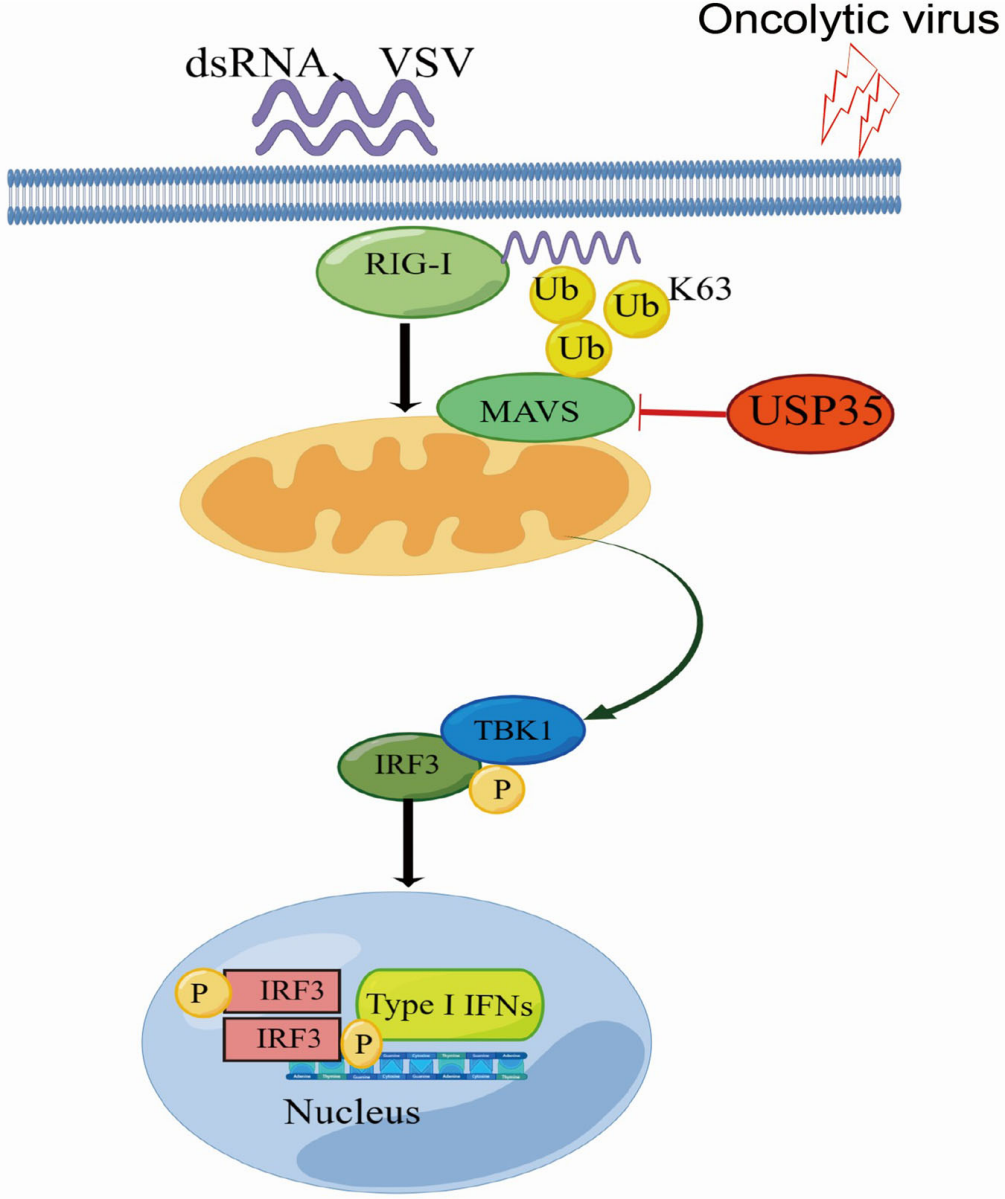
Pattern of USP35 negatively regulates the MAVS signaling pathway to attenuate type I interferon production. RNA virus is induced by exogenous RNA and cytoplasmic dsRNA, which activates the MAVS signaling pathway. However, USP35 inhibits the MAVS-TBK1-IRF3 signaling pathway through its deubiquitinating activity, thereby attenuating the production of type I interferons. The schematic was created using the Figdraw website.

Oncolytic viruses (OV) are applied in the treatment of advanced or metastatic malignant melanoma by inducing innate and adaptive immune responses to exert their anti-tumor effects [4]. Given that oncolytic virus activates the RNA signaling pathway [23], we hypothesized that the knockdown of USP35 might enhance the anti-tumor immunity triggered by OVs. To test this, a xenograft mouse model of malignant melanoma was established with B16F10 (Fig. 6A). Significant tumor shrinkage was observed in the shUSP35+OV group compared with the shNC+OV group (Fig. 6B–D), with no significant difference in body weight between the groups (Supplementary Fig. 5C). To better understand how USP35 modulates the anti-tumor ability of oncolytic virotherapy, CD4^+^/CD8^+^ T cell and macrophage type were analyzed by flow cytometric assays (Supplementary Fig. 5A, B, Supplementary Fig. 6A, B). The number of CD8^+^ T cells was significantly increased in the shUSP35+OV group compared to the shNC+OV group (Fig. 6E, F). CD8^+^ T cells were significantly increased in the shNC+OV group compared to the shNC+PBS group (Fig. 6F), consistent with the previous report showing that oncolytic viruses reprogrammed the immunosuppressed tumor microenvironment into CD8^+^ T cell-biased anti-tumor immunity [24]. No significant difference in CD4^+^ T cell infiltration was observed between the above two groups (Supplementary Fig. 5D, E). Macrophages exist in two main polarization states: M1-type, which has pro-inflammatory characteristics, and M2-type, which is typically anti-inflammatory [25]. CD206 is a specific marker for M2 macrophages [26]. Compared to the group of shNC combined with H101, the CD206 macrophages were significantly reduced in the group with shUSP35 combined with H101 (*P* < 0.05) (Fig. 6G, H, Supplementary Fig. 6A, B). Furthermore, qRT-PCR analysis revealed higher mRNA expression levels of IFNβ, CXCL10, and CCL5 in the shUSP35+OV group compared to the shNC+OV group (Supplementary Fig. 6C–H). These findings suggest that the knockdown of USP35 could enhance the anti-tumor immunity of oncolytic virotherapy by increasing CD8^+^ T infiltration, decreasing M2 macrophage polarization, and promoting the expression of key inflammatory factors such as IFNβ, CXCL10, and CCL5. In summary, we identified the deubiquitinase USP35 as a host factor modulating MAVS activity during the infection of RNA viruses. USP35 directly binds to MAVS and relies on its deubiquitinating enzyme activity. thereby inhibiting its function. (Fig. 7). Knockdown of USP35 enhances the anti-tumor immunity of malignant melanoma to oncolytic virotherapy.

## Discussion

Innate immunity serves as the first line of defense against pathogen invasion. RNA and DNA recognition are mediated by RLR-MAVS and cGAS-STING signaling pathways, respectively, which initiate the production of various cytokines such as IFN-I by activating the transcription factors IRF3 [27]. Besides its role in defending viral infection, IFN-I is also crucial for natural cancer immunosurveillance [28]. Using the luciferase reporter assay, we found that USP35 inhibits RIG-I and MAVS-mediated IFN-β reporter gene expression. Additionally, USP35 knockdown enhanced the expression of IFN-β, CXCL10, and ISG15 triggered by RNA viruses or exogenous RNA-3pRNA. A recent study suggests that USP35 interacts with STING to regulate STING activity and suppresses the antiviral type-I interferon response [17]. Our findings further underscore the role of USP35 in negatively regulating the MAVS-TBK1-IRF3 signaling pathway, and the knockdown of USP35 enhances type I interferon and inflammation-related factor expression in response to cytoplasmic RNA virus or RNA analog.

MAVS is an essential adapter protein in the RLR pathway and plays a key role in the production of type I IFN [29]. Post-translational modifications (PTMs) of MAVS are critical for for regulating itsactivity. Ubiquitination modification is a common PTM in the RLR signaling pathway. Virus infection causes K63-linked ubiquitination of MAVS in the outer mitochondrial membrane and then induces aggregation of MAVS, which is a marker of its activation [12]. The TRIM31-mediated linked ubiquitination of K63 plays an active role in the antiviral immune pathways [30]. USP18 specifically enhances the level of ubiquitination modification of MAVS and promotes ubiquitination modification and aggregation at the K63 position of MAVS [31]. In this study, Co-IP, GST-pull down and immunofluorescence experiments have shown that USP35 binds directly to MAVS. Deubiquitination experiments demonstrate that MAVS is a direct substrate of USP35 and USP35 can removes the ubiquitination modification by K63 type of MAVS through its deubiquitination activity. Besides, how USP35 modulates the activity of MAVS spatially and temporally should be fully dissected and investigated. Tumor-derived type I IFN and antigen-containing RNA can activate antigen-presenting cells (APC), mainly including DCs and macrophages. The RNA sensors in APCs sense the RNA and promote DC maturation and M1 polarization of macrophages. These cells then trigger the production of type I IFN and other proinflammatory factors that promote the anti-tumor immune microenvironment through the activation of T cells and NK cells, and inhibition of Treg and MDSC cells [32, 33]. Substantial evidence from preclinical studies supports the potential RLRs-targeted therapies in cancer treatment [34].

Oncolytic viruses selectively modify the viral genome to replicate in cancer cells and lyse cancer cells without affecting normal cells, as well as inducing an immune response in infected tumor cells [24]. Herpes simplex virus type 1 (HSV-1), a double-stranded linear DNA virus, is another oncolytic virus used in the treatment of cancer. HSV-1 infection induces mitochondrial damage and mtDNA release, which triggers cGAS/STING/IRF3 and RIG-I-MAVS signaling [23]. Recombinant human type 5 adenovirus (Ankori, H101) is a commonly used oncolytic adenovirus, which was obtained by knocking out the E1B-55KD gene fragment and part of the E3 region of wild-type human type 5 adenovirus by genetic engineering techniques [35]. It belongs to a DNA virus, but it can also be recognized by the RIG-I receptors. Specifically, the AT-rich double-stranded DNA (dsDNA) is transcribed by RNA pol III into a 5′triphosphate RNA, which further activates the RIG-I-MAVS pathway to induce the production of interferon [36].

Despite the potential of oncolytic viruses, they still face limitations including oncolytic efficacy, tumor targeting, and biosafety. Strategies to enhance combination therapies and improve treatment sensitivity are crucial for improving the survival of melanoma patients. To further evaluate the effectiveness of the combination therapy, we investigated the impact of USP35 knockdown on anti-tumor of oncolytic virotherapy using a mouse model of malignant melanoma. We observed that knockdown of USP35 combined with oncolytic virotherapy significantly inhibited the growth of B16F10. Compared to the control group, the combination therapy led to a notable increase in the number of CD8^+^T cells, a decreased M2 type macrophages, and elevated production of inflammatory factors including IFNβ. These findings suggest that targeting USP35 enhances anti-tumor immunity and boosts the efficacy of oncolytic virotherapy. There is an interaction between RNA and DNA sensing pathways in the immune response. RIG-I-like receptors can detect RNA viruses, DNA viruses, and pathogenic RNA produced during certain bacterial infections to enhance the immune response to pathogens [34]. Choi et al. showed that RIG-I may also detect DNA, suggesting that immunogenic DNA (or its transcribed RNA) may activate RLR [37]. Furthermore, the HSV-1 virus, a DNA virus discussed earlier, induces mitochondrial damage and release of mitochondrial DNA (mtDNA) upon infection, triggering both the cGAS/STING/IRF3 and RIG-I-MAVS signaling pathways [23]. These findings highlight that both RNA viruses, represented by VSV, and DNA viruses such as HSV-1, can activate the RIG-I-MAVS-IFNβ pathway via distinct mechanisms, thus contributing to tumor immunotherapy.

Zhang et al. suggested that USP35 was significantly positively correlated with “melanogenesis”, and “TORC1 signaling pathway activation”, while negatively correlated with “T cell activation”, and “macrophage activation” [38]. Previous authors have demonstrated that USP35 negatively regulates STING in the DNA signaling pathway, and our current work demonstrates that USP35 also interacts with MAVS in the RNA signaling pathway. Both DNA or RNA induction pathways stimulate the expression of type I interferons, and USP35 knockdown enhances anti-tumor immunity in response to the oncolytic virus therapy. In addition, bioinformatics analysis suggested that USP35 was elevated after immunotherapy, which also implies the importance of targeting USP35 in immunotherapy. In the future, we plan to continue to apply RIG-I agonists or LSD1 inhibitors to activate the RNA signaling pathway in combination with USP35 knockdown or knockout5 in animal models to further explore the function and mechanism. In summary, Our study identified USP35 as a novel negative regulator of the MAVS-mediated type I interferon signaling pathway in malignant melanoma cells. Targeting USP35 may offer a new therapeutic strategy to enhance anti-tumor immunity in oncolytic virotherapy.

## Supplementary information

Supplementary materials

original datas-WB

original datas-qPCR.

Supplementary Table 1

## Data Availability

The datasets analyzed for this study are in the TCGA-SKCM project (http://www.cancer.gov/tcga). The GSE database was applied to further explore the correlation between USP35 expression and malignant melanoma before and after in vivo treatment (GSE109485) (https://www.ncbi.nlm.nih.gov/geo). We used the GEPIA (cancer-pku. cn) online database to perform the gene expression and a survival prognosis analysis. The R code required to reproduce these findings cannot be shared at this time as the data also form part of an ongoing study. We used different algorithms (including xCell, QUANTISEQ, MCPcounter (R package: https://github.com/ebecht/MCPcounter), EPIC, CIBERSORTABS, and CIBERSORT) to analyze the Tumor-infiltrating Immune Cells comprehensively.
