# Supplementary material for: Deubiquitination enzyme USP35 negatively regulates MAVS signaling to inhibit anti-tumor immunity
Source: Cell Death Dis. 2025 Feb 27;16(1):138. doi: 10.1038/s41419-025-07411-8 (PMC11868397; doi:10.1038/s41419-025-07411-8)
Supplement: Supplementary file 1 — Supplementary materials [file 41419_2025_7411_MOESM1_ESM.pdf]

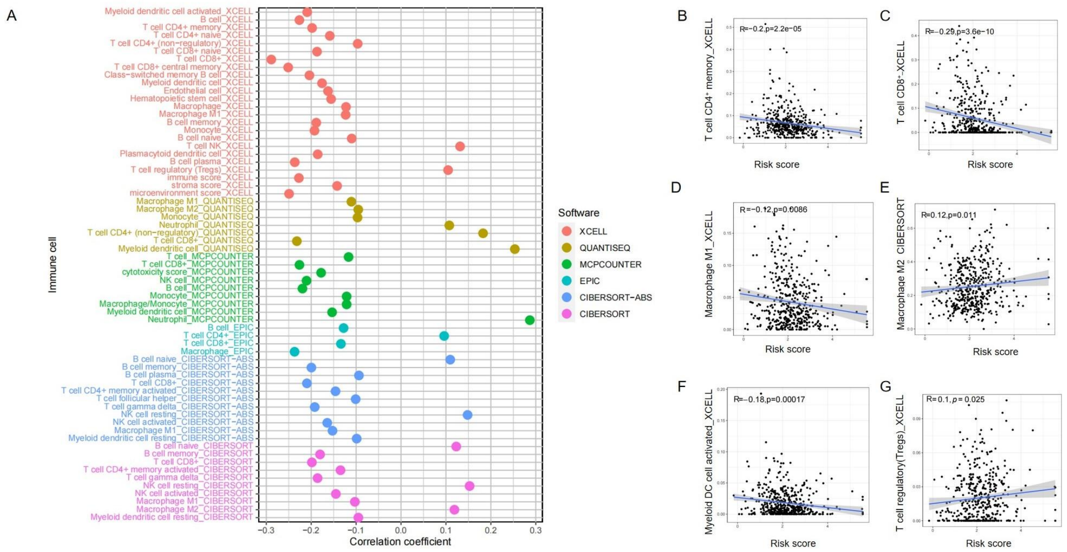

Supplementary Figure S1 Correlation between USP35 expression and tumor-infiltrating immune cells. Correlation coefficients greater than 0 indicate a positive correlation, and less than 0 indicate a negative correlation. (A) Bubble diagram of the correlation between USP35 expression and immune cells. (B-G) Correlation analysis of USP35 expression with CD4^{+}, CD8^{+} T cells, M2 macrophages , M1 macrophages, dendritic cells (DCs) and Treg cells.

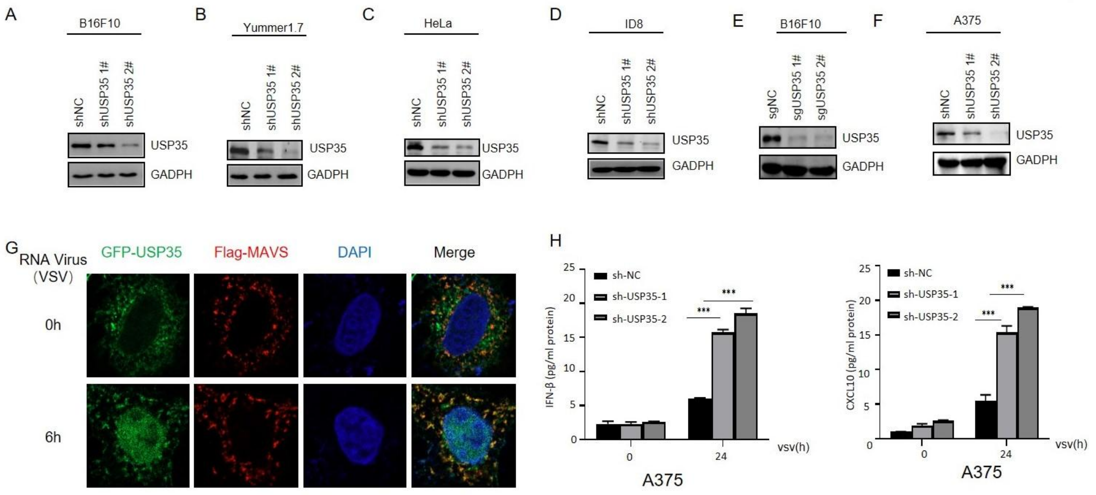

Supplementary Figure S2 USP35 knockdown and overexpression analysis in various cell lines. (A-D, F) B16F10, Yummer1.7, HeLa, ID8, and A375 were stably infected with shRNA targeting USP35. Western blot (WB) was performed to assess the knockdown efficiency of USP35 in these cells. (E)In sgUSP35-B16F10-Cas9 ,

Western blotting was used to detect the knockout efficiency of USP35. (G) Immunofluorescence staining was performed to observe the co-localization of GFP-USP35 and Flag-MAVS with or without VSV infection for 6 h. (H) Cell culture media from A375 with shUSP35 or shNC treatment were collected at 0 h and 24 h post RNA virus infection. ELISA was used to measure the levels of human IFNβ and CXCL10 in the supernatants.

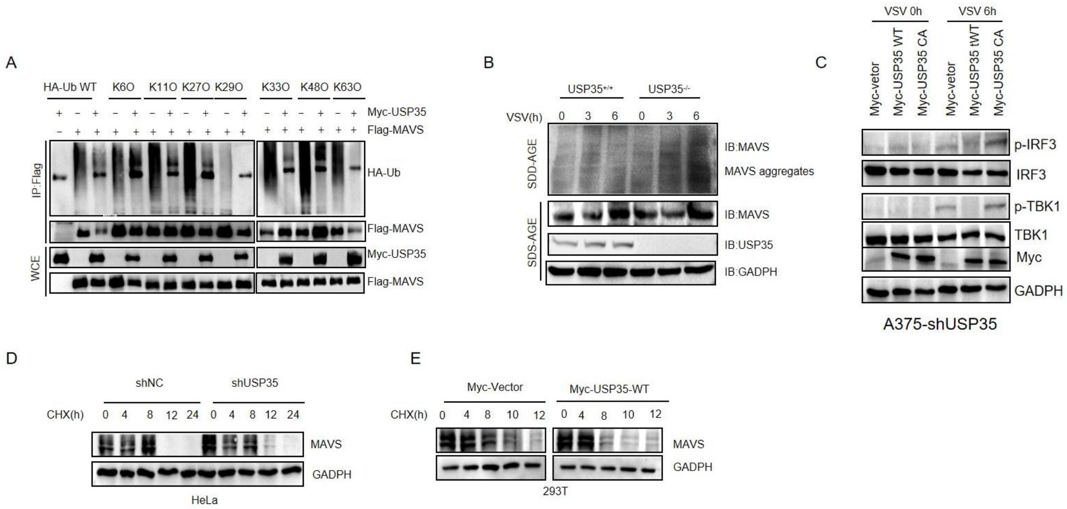

Supplementary Figure S3 The mechanism of USP35 regulates MAVS. (A) Denature-IP (with anti-HA) and immunoblot analysis (with anti-Flag, anti-HA, or anti-Myc) of HEK293T transfected with indicated plasmids. (B) SDD-AGE analysis (top) and SDS-PAGE (below) of the aggregation of MAVS in USP35$^{+/+}$ and USP35$^{-/-}$ HeLa infected for 0, 6, and 8h (above lanes) with VSV. (C) In shUSP35 cells rescued with Myc-Vector, Myc-USP35 WT, and Myc-USP35 CA, samples were collected after viral infection, and the protein expression of p-TBK1, p-IRF3, Myc and GAPDH, TBK1, and IRF3 were detected by WB. (D) Immunoblot analysis of MAVS and GAPDH in shNC and shUSP35 of HeLa treated for 0-24 h in the presence or absence of cycloheximide (100μg/ml) (E) Immunoblot analysis of MAVS and GAPDH in 293T with Myc-Vector and Myc-USP35 treated for 0-24 h in the presence or absence of cycloheximide (100μg/ml).

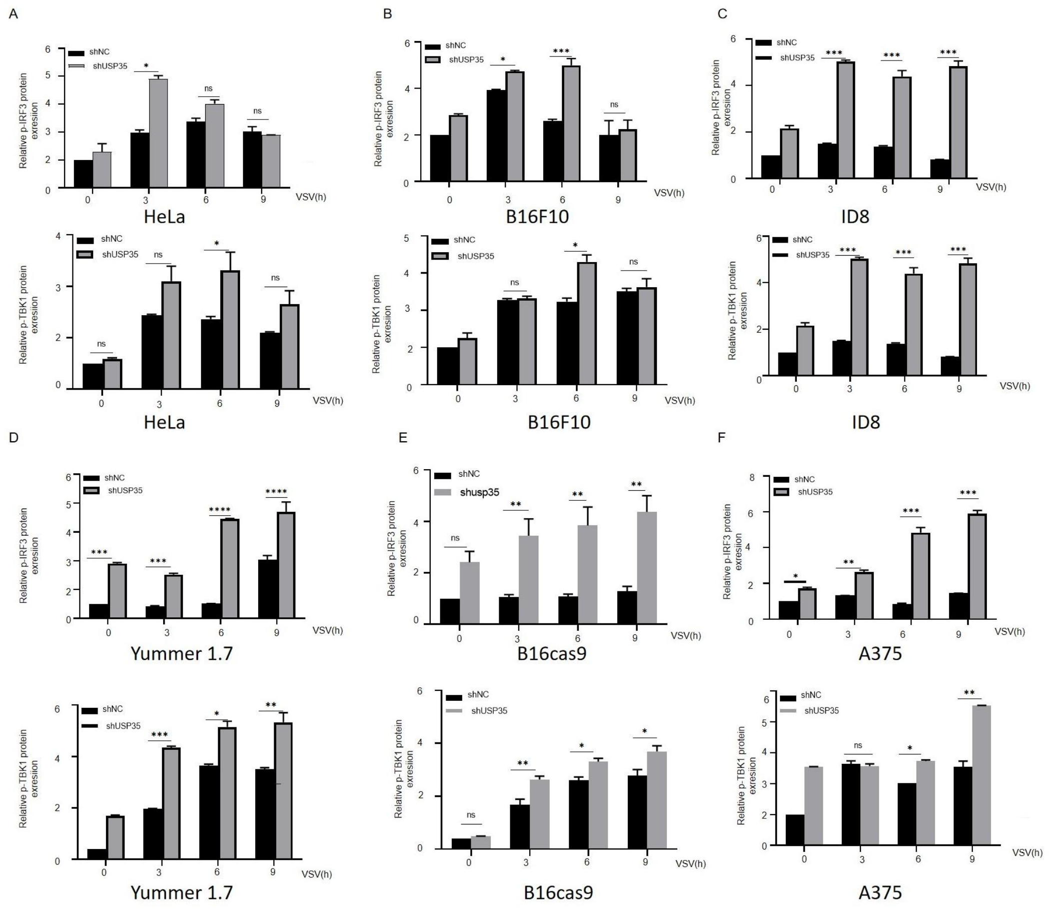

Supplementary Figure S4 Presentation of protein grayscale values for each data set in Figure 5. Grayscale values corresponding to the protein bands shown in Figure 5 are presented for each dataset. The grayscale values represent the relative protein expression levels for pTBK1, pIRF3 as quantified by Western blot analysis. The significance statistics method is Two-way ANOVA, where *P <0.05, **P <0.01, **P <0.01, ns is not statistically different,

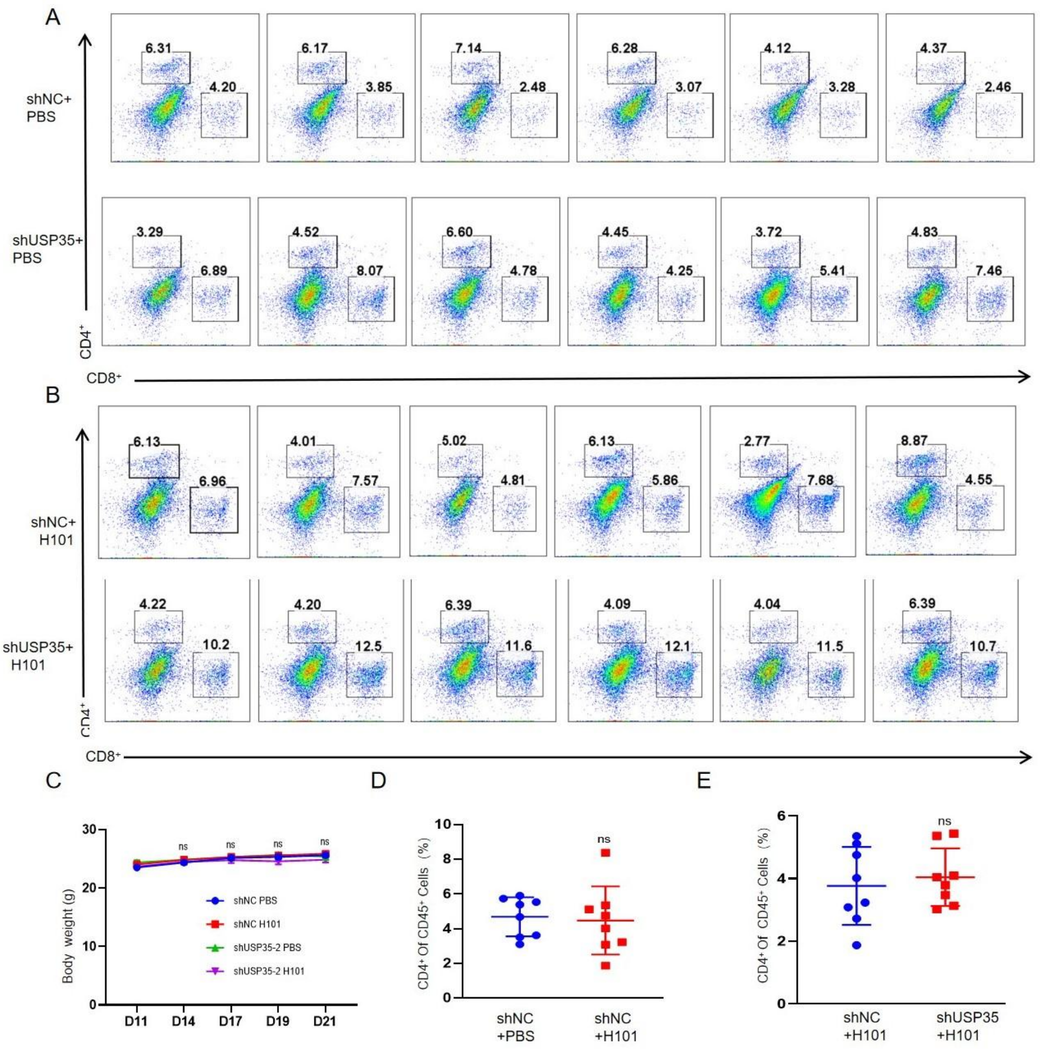

Supplementary Figure S5 Cellular analysis and immune cell profiling in mouse models. (A-B) Flow cytometry data from one mouse per group (8 mice per group) is presented. The gates were drawn in the following order: first, tumor-infiltrating immune cells were selected, followed by CD45-positive cells, and then CD4+ T and CD8+ T cells were drawn from the CD45+ population. The proportion of each cell cluster was calculated for the first mouse, and this gating strategy was applied to the remaining mice in the group. (C) Body weight of mice in each experimental group was recorded. (D) The proportion of $CD4^{+}$ T cells in the shNC+PBS group versus the shUSPNC+H101 group. (E) The proportion of $CD4^{+}$ T cells in the shNC+H101 group versus the shUSP35+H101 group.

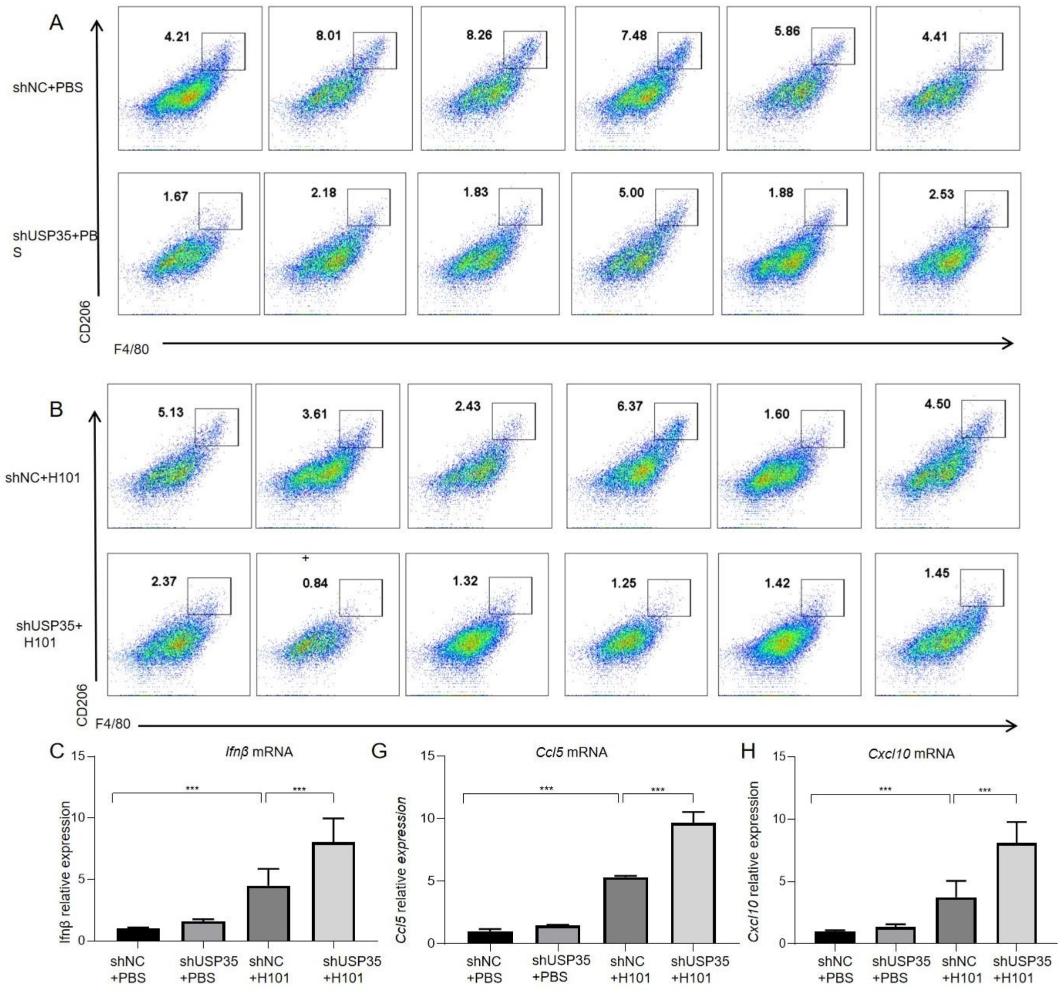

Supplementary Figure S6 Cellular analysis and gene expression profiling in mouse models. (A-C) Flow cytometry analysis of one mouse per group (8 mice per group) is shown. The gates were drawn in the following order: tumor-infiltrating immune cells were circled first, followed by CD45-positive cells, and then F480+ and CD206+ cells within the CD45+ population. The proportion of each cell cluster in one mouse was determined first, and this gating strategy was applied to the remaining mice in the group. (D-F) Quantitative PCR (qPCR) analysis of mRNA expression levels of IFNβ, CXCL10, and CCL5 in mouse tissues from each experimental group. Data are presented as Mean ± s.d. Statistical significance was determined using Student's t-test, where $*P < 0.05$, $***P < 0.001$, and ns indicates no statistical difference.qPCR detection of mRNA expression of IFNβ, CXCL10, and CCL5 in

mouse tissues of each group. The data were shown as Significance statistics were performed by Student's t-test, *$P < 0.05$, ***$P < 0.001$, ns, no statistical difference.
