# Supplementary material for: Deubiquitination enzyme USP35 negatively regulates MAVS signaling to inhibit anti-tumor immunity
Source: Cell Death Dis. 2025 Feb 27;16(1):138. doi: 10.1038/s41419-025-07411-8 (PMC11868397; doi:10.1038/s41419-025-07411-8)
Supplement: Supplementary file 2 — original datas-WB [file 41419_2025_7411_MOESM2_ESM.pptx]

## Slide 1
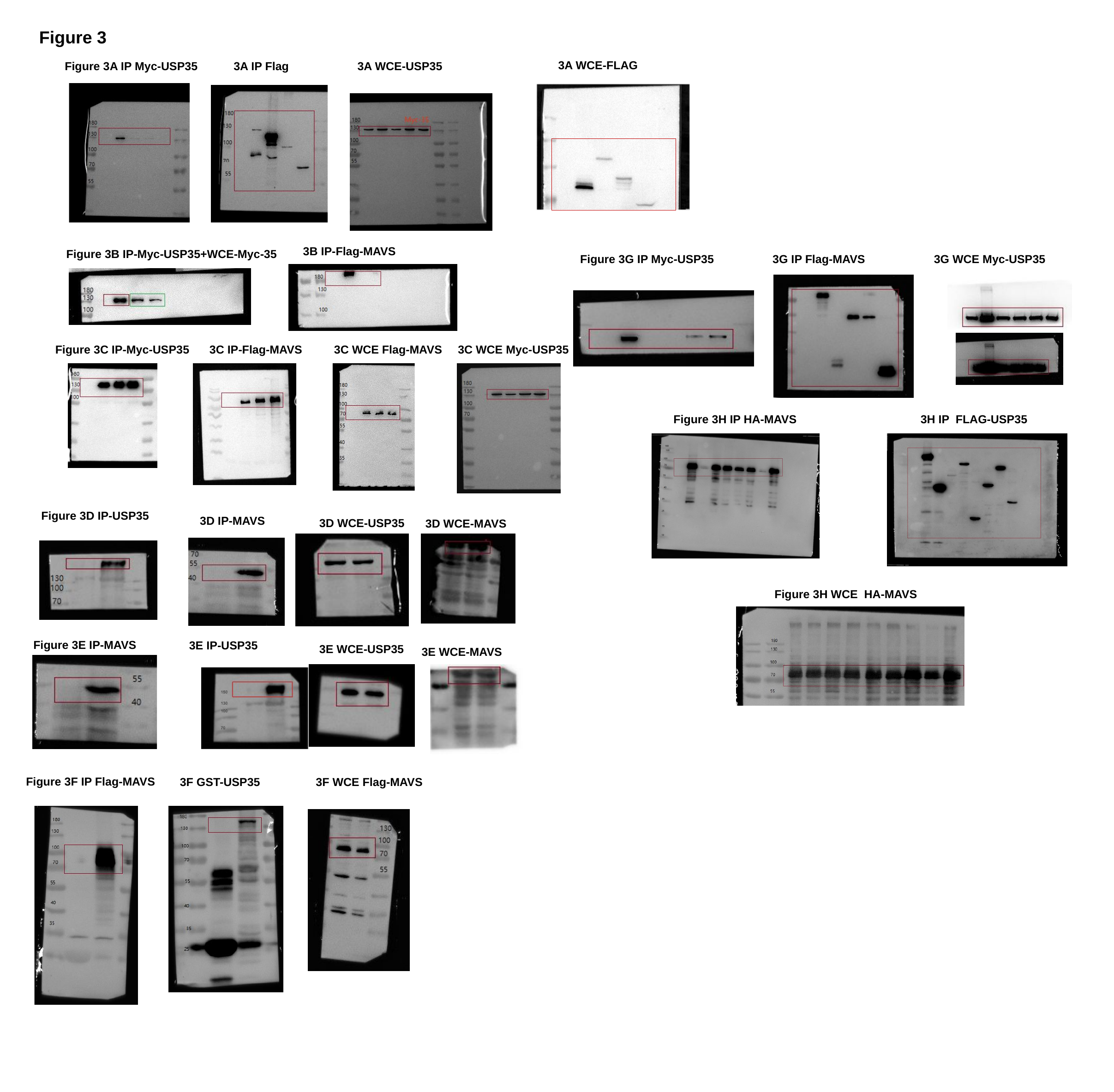

Figure 3
3A WCE-FLAG
Figure 3A IP Myc-USP35
3A IP Flag
3A WCE-USP35
 3B IP-Flag-MAVS
Figure 3B IP-Myc-USP35+WCE-Myc-35
Figure 3G IP Myc-USP35
 3G IP Flag-MAVS
 3G WCE Myc-USP35
Figure 3H IP HA-MAVS
3H IP FLAG-USP35
Figure 3H WCE HA-MAVS
Figure 3C IP-Myc-USP35
3C IP-Flag-MAVS
3C WCE Flag-MAVS
3C WCE Myc-USP35
Figure 3D IP-USP35
3D IP-MAVS
3D WCE-USP35
3D WCE-MAVS
Figure 3E IP-MAVS
3E IP-USP35
3E WCE-USP35
3E WCE-MAVS
Figure 3F IP Flag-MAVS
3F GST-USP35
3F WCE Flag-MAVS

## Slide 2
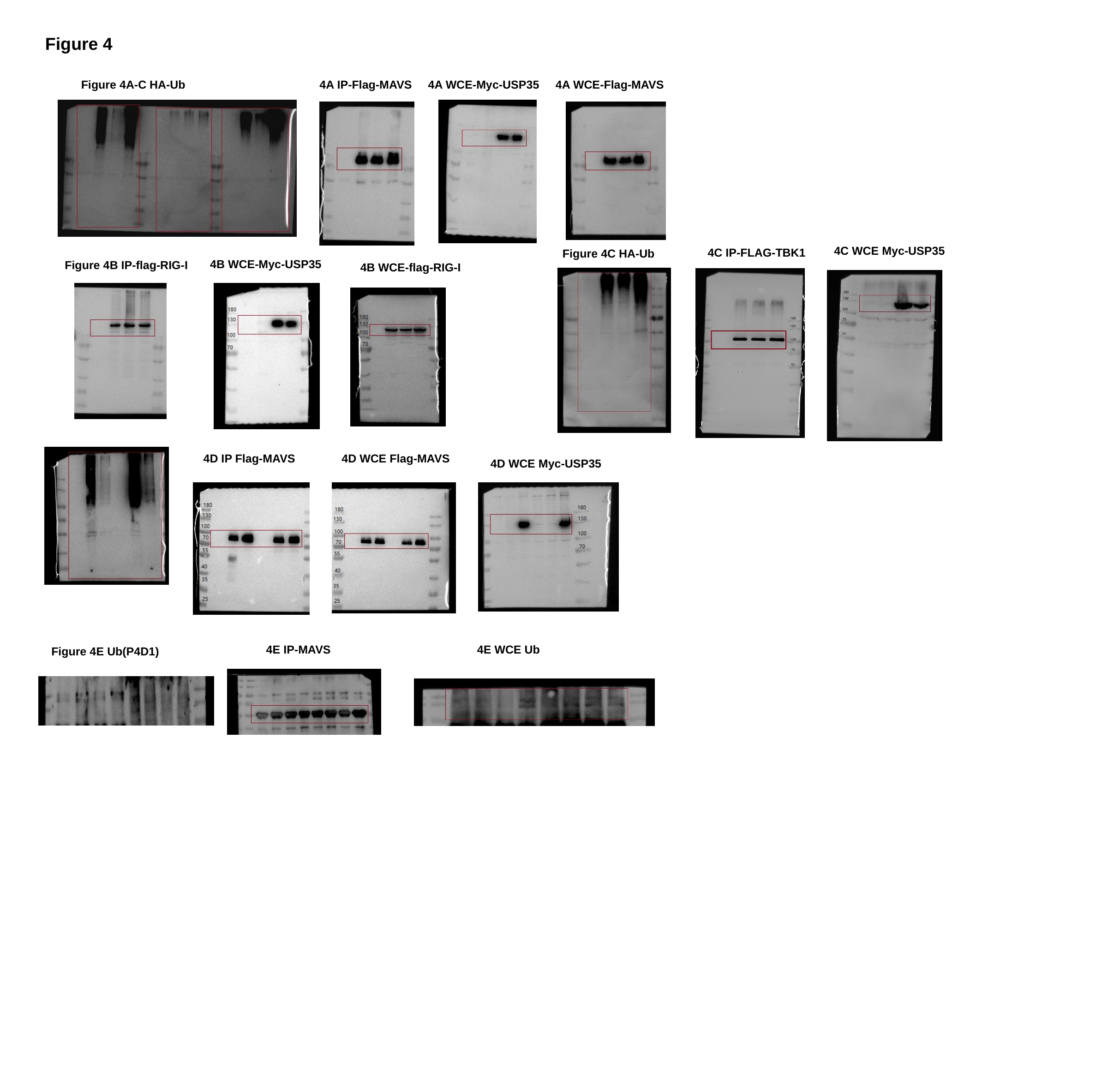

Figure 4
Figure 4A-C HA-Ub
 4A IP-Flag-MAVS
 4A WCE-Myc-USP35
 4A WCE-Flag-MAVS
4C WCE Myc-USP35
4C IP-FLAG-TBK1
Figure 4C HA-Ub
4B WCE-Myc-USP35
Figure 4B IP-flag-RIG-I
 4B WCE-flag-RIG-I
Figure 4D HA-Ub
 4D IP Flag-MAVS
 4D WCE Flag-MAVS
 4D WCE Myc-USP35
4E IP-MAVS
4E WCE Ub
Figure 4E Ub(P4D1)

## Slide 3
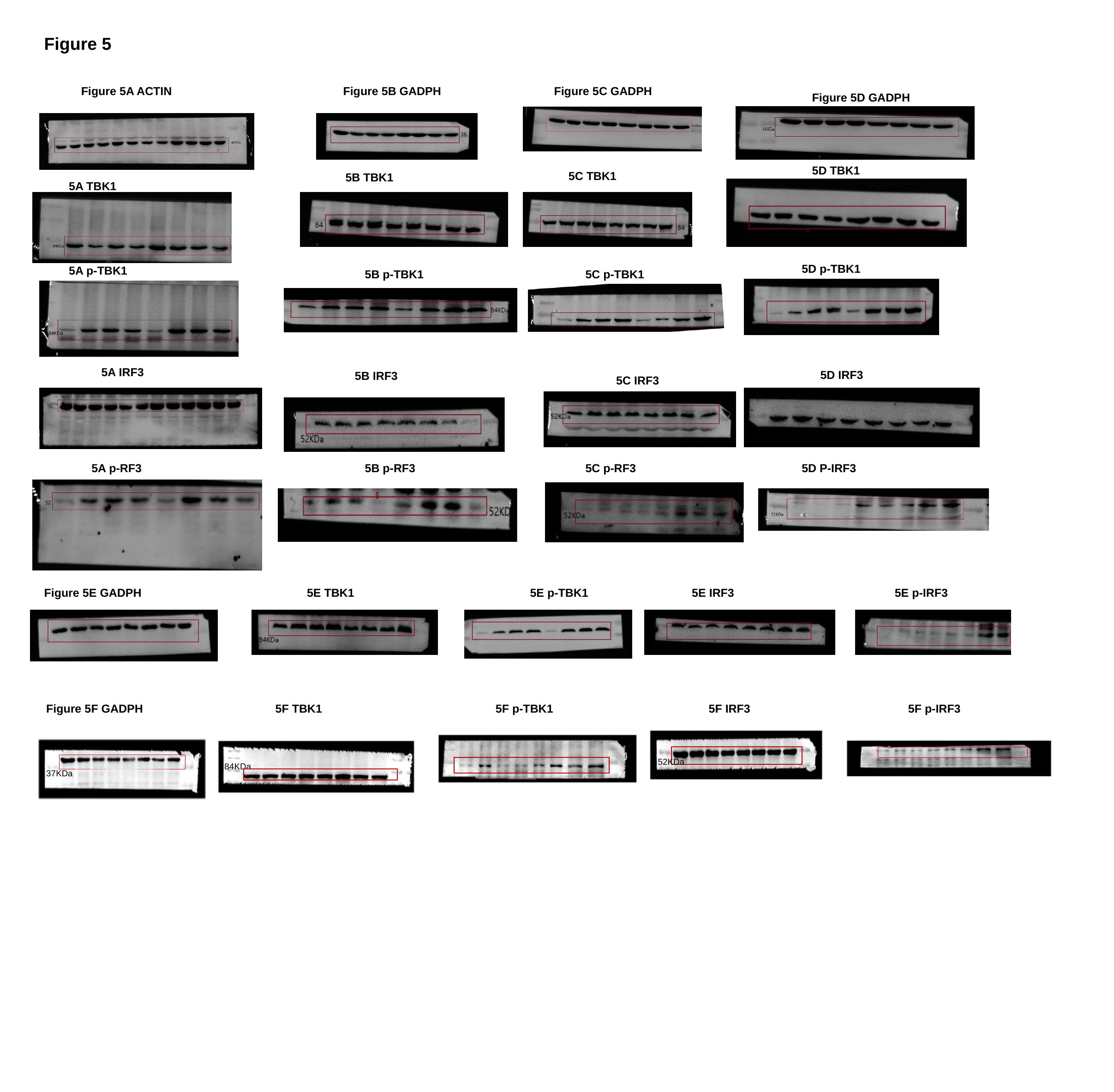

Figure 5
Figure 5A ACTIN
Figure 5B GADPH
Figure 5C GADPH
Figure 5D GADPH
5D TBK1
5C TBK1
5B TBK1
5A TBK1
5D p-TBK1
5A p-TBK1
5B p-TBK1
5C p-TBK1
5A IRF3
5D IRF3
5B IRF3
5C IRF3
5A p-RF3
5B p-RF3
5C p-RF3
5D P-IRF3
Figure 5E GADPH
5E TBK1
5E p-TBK1
5E IRF3
5E p-IRF3
Figure 5F GADPH
5F TBK1
5F p-TBK1
5F IRF3
5F p-IRF3
52KDa
37KDa
84KDa

## Slide 4
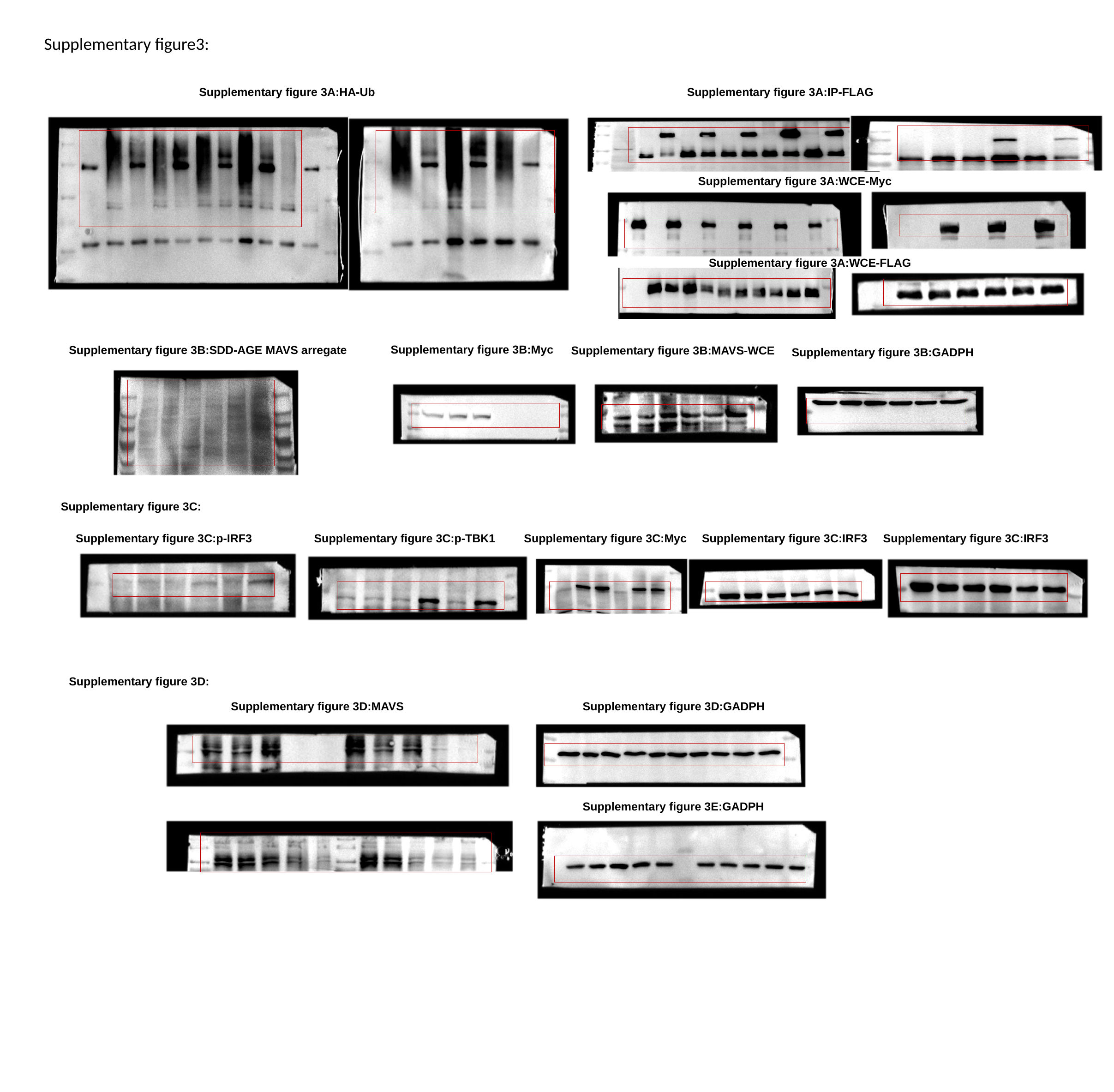

Supplementary figure3:
Supplementary figure 3A:HA-Ub
Supplementary figure 3A:IP-FLAG
Supplementary figure 3A:WCE-Myc
Supplementary figure 3A:WCE-FLAG
Supplementary figure 3B:Myc
Supplementary figure 3B:SDD-AGE MAVS arregate
Supplementary figure 3B:MAVS-WCE
Supplementary figure 3B:GADPH
Supplementary figure 3C:
Supplementary figure 3C:p-IRF3
Supplementary figure 3C:p-TBK1
Supplementary figure 3C:Myc
Supplementary figure 3C:IRF3
Supplementary figure 3C:IRF3
Supplementary figure 3D:
Supplementary figure 3D:MAVS
Supplementary figure 3D:GADPH
Supplementary figure 3E:GADPH
